# The Role of Bioactive Compounds in Immunonutrition

**DOI:** 10.3390/nu16203432

**Published:** 2024-10-10

**Authors:** Fernando Rivero-Pino, Sergio Montserrat-de la Paz

**Affiliations:** 1 Department of Medical Biochemistry, Molecular Biology and Immunology, School of Medicine, University of Seville, Av. Sanchez Pizjuan s/n, 41009 Seville, Spain; 2 Instituto de Biomedicina de Sevilla, IBiS, Hospital Universitario Virgen del Rocio, CSIC, University of Seville, 41013 Seville, Spain

The link between diet and immune function is a growing area of interest, recognized not only by the scientific community but also by global health organizations, such as the World Health Organization (WHO). As research continues to unveil the complex interactions between nutrients, bioactive compounds, and the immune system, it is becoming increasingly clear that what we consume plays a pivotal role in shaping our body’s defense mechanisms. This Special Issue, “The Role of Bioactive Compounds in Immunonutrition”, explores these intricate relationships and highlights the potential of dietary components in modulating immune responses, reducing inflammation, and improving health outcomes across various disease states. This Special Issue features a total of twelve papers, including three systematic reviews, one narrative review, and eight original research articles, which collectively explore the diverse sources and effects of bioactive compounds derived from food, mainly plants, on immune function.

The systematic reviews cover a range of plant-derived products and their bioactive properties. One review evaluates the efficacy and safety of East Asian herbal medicine combined with ultraviolet B therapy for psoriasis [1]. These authors collected randomized controlled trials from ten databases in Korea, China, and Japan, leading to an analysis of 3521 patients from 40 studies, from which promising but not conclusive results could be drawn. Another systematically examines the biological functions, mechanistic actions, and therapeutic potentials of chlorogenic acid [2], a compound found in coffee and many fruits and vegetables. As a bioactive compound, chlorogenic acid could offer benefits against various pathological conditions, particularly chronic metabolic and age-related disorders. Its key functions include neuroprotection in neurodegenerative diseases and diabetic neuropathy, anti-inflammatory, antioxidant, antimicrobial, cardioprotective, and anti-tumor activities, and protective effects on skin, liver, kidney, and metabolic health. It modulates anti-inflammatory and antioxidant pathways, such as NF-kB and Nrf2, and enhances AMPK signaling to maintain metabolic balance. However, according to the authors, there is still a need for further studies to identify its specific targets, improve its bioavailability, and validate its clinical efficacy. On the other hand, the potential of non-digestible carbohydrates was also systematically reviewed [3]. In this case, from a total of 101 articles retrieved, the authors elaborated on studies involving by-products with experimental data and green or GRAS extraction methods that optimized instrumental variables through experimental designs; they also reviewed in vitro or in vivo studies that analyzed microbiota modulation using dietary fibers such as pectin, inulins, and alginates and explored their combinations with phenolic compounds. In addition to these systematic reviews, the immunomodulatory properties of food-derived peptides are also reported as part of this Special Issue [4]. However, according to the authors, promising results are limited, and some studies did not show observable physiological changes. When responses were detected, they often did not address key parameters, making it difficult to clearly establish the immunomodulatory properties with the current evidence. It was concluded that well-designed clinical trials are necessary to better assess the role of protein hydrolysates in immunonutrition.

Among the eight research articles, the main interests were olive oil and related products and the evaluation of microbiota. First, in vitro studies focused on olive products, such as assessing the modulation of primary human neutrophils activated by beta-amyloid using dietary phenols from virgin olive oil [5]. In Alzheimer’s disease, beta-amyloid (Aβ1-42) acts as an early trigger, causing synaptic and cognitive impairments. Virgin olive oil has been linked to a reduced risk of immune–inflammatory disorders, but the impact of its phenolic fraction (PF) on neuroinflammatory processes in neutrophils was previously unknown. This study examined how PF affects Aβ1-42-stimulated primary human neutrophils, focusing on gene expression, surface marker changes, and the release of pro-inflammatory and chemoattractant mediators. The results showed that PF significantly down-regulated pro-inflammatory cytokine gene expression and prevented neutrophil activation. In a similar line, olive leaf phenolic compounds were studied for their gastrointestinal stability in vitro based on co-administration with non-digestible carbohydrates [6]. These authors also posit that olive leaves are a valuable source of bioactive compounds, particularly phenolic compounds with known antioxidant, anti-inflammatory, and immunomodulatory properties, but their application is hampered by their instability under gastrointestinal conditions. A spray-drying encapsulation process using inulin as the encapsulating agent for a phenolic-rich olive leaf extract was optimized. Afterwards, the behavior of the free extract, its co-administration with the encapsulating agent, and the optimized microencapsulated formulation through an in vitro gastrointestinal digestion process using the INFOGEST protocol was evaluated. The results showed that the free extract underwent significant degradation during digestion. In contrast, when co-administered with inulin, the degradation of the extract was reduced. The protective effect observed with inulin co-administration was comparable to that of the microencapsulated formulation. These two articles show the applicability and potential health effects of phenols from olives. In addition to these in vitro studies, evidence on how *n*-3 and *n*-6 polyunsaturated fatty acids modulate macrophage–myocyte inflammatory crosstalk and improve myocyte insulin sensitivity was also reported [7]. In this context, it was suggested that dietary long-chain *n*-3 and *n*-6 PUFAs could be an adequate strategy to mitigate inflammatory and metabolic dysfunction in obesity.

Recent animal studies have also explored innovative approaches for treating chronic conditions by targeting underlying biological mechanisms. One study investigated the use of Erigeron annuus extract (EAE) for managing atopic dermatitis (AD), a persistent inflammatory skin condition influenced by genetic, immunological, and environmental factors. The research demonstrated that EAE, described as potentially beneficial for its anti-inflammatory, cytoprotective, and antioxidant properties, effectively reduced oxidative stress in vitro by lowering malondialdehyde levels and increasing superoxide dismutase activity in human keratinocytes. In vivo tests with an AD mouse model showed that EAE treatment led to decreased ear epidermal thickness and reduced scratching behavior, suggesting its potential to ameliorate AD symptoms by modulating the Nrf2/HO-1 signaling pathway [8]. Meanwhile, another study focused on spinal cord injury (SCI) and the gut–central nervous system axis, revealing that interleukin-13 (IL-13) could enhance functional recovery by altering gut microbiota composition. Systemic rIL-13 treatment in a mouse model of SCI resulted in significant changes in microbial communities, including an increased abundance of Clostridiales vadin BB60 and Acetitomaculum and improvements in functional recovery. This suggests that IL-13 might indirectly support recovery by modifying gut microbiota, potentially paving the way for novel SCI therapies [9].

Human studies play a crucial role in advancing the field of immunonutrition by providing evidence of how specific dietary components can influence immune function and overall health. For example, a study investigating the Lactococcus lactis strain Plasma (LC-Plasma), a lactic acid bacterium known for its effects on plasmacytoid dendritic cells (pDCs), demonstrated its potential in alleviating exercise-induced fatigue. Participants who consumed LC-Plasma showed increased pDC maturation markers and fewer cumulative days of fatigue compared to those taking a placebo, highlighting the strain’s capacity to enhance immune function and manage fatigue during prolonged physical exertion [10]. Similarly, research on heat-treated Lactiplantibacillus plantarum nF1 (HT-nF1) underscored its benefits on immune health. In a randomized, double-blind, placebo-controlled study, HT-nF1 supplementation led to significant increases in immune markers such as interleukin-12 and immunoglobulins, particularly in individuals with initially low secretory IgA and natural killer cell activity [11]. These studies collectively illustrate the importance of human clinical trials in validating the immunomodulatory effects of specific probiotic strains, offering insights into their potential as functional health food ingredients and their role in improving immune function and managing fatigue.

Finally, sociodemographic studies are vital for understanding the factors that influence dietary behaviors and health outcomes, particularly during critical transitions such as the shift from adolescence to university life. In this regard, the determinants and barriers affecting the diversity of fruit and vegetable (FV) consumption among undergraduate students was reported [12]. Conducted as a cross-sectional study with 542 students from Chulalongkorn University in Bangkok, Thailand, the research assessed FV intake in relation to sociodemographic factors and cooking methods. The results indicated a strong preference for tropical fruits with inedible peels (88.2%) and Brassicaceae vegetables (91.0%), while citrus fruits (19.7%) and Fabaceae vegetables (43.7%) were consumed less frequently. Sociodemographic factors such as gender, living arrangements, BMI, and academic year significantly impacted FV consumption. Specifically, male students, those living independently, those with a lower BMI, and students in advanced academic years exhibited lower FV intake. Additionally, a lower quality of life was associated with a higher proportion of students not consuming vegetables. The study identified busy lifestyles as a barrier to fruit intake and taste preferences as a key motivator for fruit consumption. These findings highlight the need for targeted strategies and environmental changes within the university setting to promote a diverse and healthy FV diet among students.

This Special Issue’s breadth—from systematic reviews of herbal medicine to research on specific compounds like chlorogenic acid and probiotics—demonstrates a well-rounded approach to understanding how different dietary elements can affect immune responses. Together, these studies highlight the potential of food-derived bioactive compounds, ranging from polyunsaturated fatty acids, probiotics, and plant extracts to food-derived peptides, in modulating immune function and improving overall health outcomes across various contexts [13,14,15]. However, translating these findings into practical dietary recommendations requires further exploration [16,17]. For instance, while some studies show potential, the variability in individual responses and the need for standardized dosages highlight the importance of personalized nutrition approaches. Future research should focus on large-scale, longitudinal studies to validate the clinical efficacy of these compounds and determine optimal intake levels for different populations. Additionally, investigating the interactions between various dietary components and their combined effects on immune health could provide a more holistic understanding [18,19,20]. Developing guidelines based on this comprehensive evidence could lead to actionable recommendations for enhancing immune function through diet, ultimately contributing to better public health strategies and personalized nutrition plans.

The role of bioactive compounds in immunonutrition is an emerging field of great significance, as it highlights the intricate interplay between diet and immune function. Nutrients such as vitamins, minerals, polyphenols, and fatty acids have demonstrated potential in modulating immune responses, either by enhancing immunity or by reducing chronic inflammation, which is pivotal in preventing diseases. These components, such as vitamins, are essential for maintaining the integrity and functionality of immune cells [21], while polyphenols, such as flavonoids, exert anti-inflammatory and antioxidant effects. Omega-3 fatty acids are particularly beneficial for regulating inflammation, improving immune cell activity, and potentially reducing the risk of autoimmune conditions [22,23]. Furthermore, the gut microbiota, significantly influenced by diet, plays a crucial role in shaping immune responses. Bioactive compounds like prebiotics and probiotics can enhance the gut barrier and modulate immune responses, suggesting a direct link between dietary components and immune health. Given the ongoing challenges posed by infectious diseases and autoimmune disorders, a deeper understanding of how bioactive compounds can support immune resilience is crucial [24,25,26].
